# Circulating Prokineticin 2 Levels Are Increased in Children with Obesity and Correlated with Insulin Resistance

**DOI:** 10.1155/2021/6630102

**Published:** 2021-04-04

**Authors:** Han Wang, Yanjun Jia, Xiaoyan Yu, Li Peng, Chunfeng Mou, Zhixin Song, Dapeng Chen, Xiaoqiang Li

**Affiliations:** ^1^Department of Clinical Laboratory, Children's Hospital of Chongqing Medical University, National Clinical Research Center for Child Health and Disorders, Ministry of Education Key Laboratory of Child Development and Disorders, Chongqing Key Laboratory of Pediatrics, Chongqing Key Laboratory of Child Health and Nutrition, Chongqing 400014, China; ^2^Department of Endocrinology, The Second Affiliated Hospital, Chongqing Medical University, Chongqing 400010, China; ^3^Department of Nuclear Medicine, Children's Hospital of Chongqing Medical University, Chongqing 400014, China

## Abstract

**Objective:**

Prokineticin 2 (PK2) has been shown to regulate food intake, fat production, and the inflammation process, which play vital roles in the pathogenesis of obesity. The first aim of this study was to investigate serum PK2 levels in children with obesity and normal-weight children. The second aim was to compare the levels of PK2 between children with obesity, with and without nonalcoholic fatty liver disease (NAFLD).

**Methods:**

Seventy normal-weight children and 91 children with obesity (22 with NAFLD) were recruited. Circulating PK2, IL-6, and TNF-*α* were measured by enzyme-linked immunosorbent assays. Anthropometric and biochemical measurements related to adiposity, lipid profile, and insulin resistance were examined for all participants.

**Results:**

Serum PK2 was significantly higher in children with obesity than in the normal-weight controls. Circulating PK2 levels were not different between the patients with and without NAFLD. Circulating PK2 was positively correlated with BMI, BMI z-score, insulin, glucose, HOMA-IR, total cholesterol, low-density lipoprotein cholesterol, alanine aminotransferase, and gamma-glutamyl transpeptidase. Binary logistic regression revealed that the odds ratios for obesity were significantly elevated with increasing PK2.

**Conclusions:**

PK2 was strongly associated with obesity, and it may also be related to metabolic disorders and insulin resistance. This trial is registered with ChiCTR2000038838.

## 1. Introduction

Obesity is a global problem and its prevalence is increasing in children [1]. A high percentage of children with obesity tend to become adults with obesity [2]. Moreover, overweight or obesity in childhood is an important risk factor for metabolic diseases such as type 2 diabetes (T2DM), hypertension, and nonalcoholic fatty liver disease (NAFLD) in adulthood. NAFLD is a common serious complication of childhood obesity influencing up to 50% of children with obesity [3, 4]. It covers various hepatic lesions, from simple to progressive steatosis and inflammation, fibrosis, cirrhosis, and eventually hepatocellular carcinoma [5]. Therefore, biomarkers for obesity are of great importance for early diagnosis and implementation of preventive strategies to decrease the burden associated in adulthood.

Obesity is usually associated with a low-level chronic inflammatory state, marked by an increase in systemic inflammatory markers [6]. The deposition of superfluous nutrients in adipocytes or tissues could induce the production of inflammatory regulatory factors, such as proinflammatory factors (interleukin- (IL-) 1, IL-6, tumor necrosis factor- (TNF-) *α*, monocyte chemoattractant protein 1 (MCP-1)) and anti-inflammatory factors (adiponectin, IL-4, IL-13, IL-10) released into the blood circulation, which could regulate the inflammatory response in the body [7]. In patients with insulin resistance and obesity, the production and expression of IL-6, TNF-*α*, and MCP-1 are significantly increased [8]. Meanwhile, low-level chronic inflammation is thought to contribute significantly to the development of obesity-related pathologies including insulin resistance, dyslipidemia, T2DM, and hypertension [9–11]. Thus, clarifying the relationship between obesity and inflammation is helpful to provide a theoretical basis for the prevention, diagnosis, and treatment of obesity and obesity-related diseases.

Prokineticin 2 (PK2) is a polypeptide molecule containing 81 amino acids and belongs to the prokineticin family [12]. PK2 is widely distributed in mammalian tissues [13] and involved in diverse physiological effects such as angiogenesis [14–16], hematopoiesis [17], and neurogenesis [18]. Recent studies have confirmed that PK2 is involved in the inflammatory process. PK2 could induce the migration of mouse macrophages and establish a proinflammatory phenotype to stimulate the production of lipopolysaccharides and induce proinflammatory cytokines (IL-1 and IL-12) and reduce the expression of anti-inflammatory cytokines IL-10 [19]. PK2 was found in high levels in inflammatory mouse tissues with infiltrating neutrophils and could modulate pain caused by inflammation [20]. However, the relationship between PK2 and childhood obesity in which inflammation plays an important role still remains poorly understood.

PK2 is also involved in regulating food intake and fat production. Intraventricular injection of PK2 could effectively inhibit the food intake of rats, while anti-PK2 antibody could increase the food intake, indicating that PK2 is a neuropeptide associated with anorexia [21]. Peripheral injection of PK2 in a mouse model of obesity also reduced the mice's food intake and body weight through anorexia effect mediated by the brain stem [22]. In addition, PK2 was highly expressed in the white adipose tissue of people with obesity, suggesting that PK2 may be an adipose factor [23]. PK2-knockout mice and populations with homozygous inactivated mutations of PK2 or PK2 receptor both became obese [22, 24]. However, the research conclusion of PK2 in a population with obesity-related metabolic diseases is contradictory [25, 26]. Moreover, the rhythmicity of physiological and behavioral functions driven by the main circadian clock located in the suprachiasmatic nucleus of the anterior hypothalamus was damaged in mice with impaired PK2 pathway [27, 28]. As we know, disruption of circadian clock results in dyslipidemia, insulin resistance, and obesity [29]. Thus, the association among PK2, obesity, and obesity-related metabolic diseases needs further investigation.

NAFLD is closely associated with obesity. However, to our best knowledge, the characteristics of circulating PK2 in children with obesity with/without NAFLD have not yet been reported. Therefore, the first objective of this study was to investigate serum PK2 levels and the potential relationship between PK2 and metabolic parameters in children with obesity and normal-weight children. The second objective was to compare the levels of PK2 between children with obesity, with and without NAFLD.

## 2. Materials and Methods

### 2.1. Subjects

In all, 161 children, including 91 children with obesity (60 boys and 31 girls) and 70 age-matched healthy children (46 boys and 24 girls), were recruited in the current study. The control subjects were recruited from routine medical check-up at the Department of Physical Examination Center in Children's Hospital of Chongqing Medical University. Children with obesity were defined as those having a body mass index (BMI) > 95th percentile of the appropriate age and sex, according to BMI growth reference for Chinese children aged 0–18 years [30]. BMI is calculated as weight in kilograms divided by the square of the height in meters. BMI was converted into a BMI z-score according to the least mean squares method [30, 31]. The exclusion criteria included the following: genetic or endocrine obesity; use of metabolic drugs such as thyroxine tablets, dexamethasone, metformin; hepatic or renal disease; cancers; recent infection; and/or any medical or mental disease that would interfere with the study participation. None of the subjects or their legal guardians reported significant sleep and circadian rhythm abnormalities.

NAFLD was diagnosed based on increased echogenicity on ultrasound compatible with fatty infiltration of the liver with or without elevated alanine aminotransferase (ALT) levels [32]. Children with obesity were divided into two groups: those with NAFLD (*n* = 22) and those without (*n* = 69) according to the liver brightness on ultrasonography. The present study was conducted in accordance with the Declaration of Helsinki and approved by the Institutional Review Board of Children's Hospital of Chongqing Medical University and was registered at ChiCTR2000038838.

### 2.2. Clinical and Biochemical Measurements

All individuals participated in the series of anthropometric and body composition examination after 10–12 h of overnight fasting. Age, height, and weight were recorded by trained staff. Blood samples were obtained from all individuals before breakfast and were centrifuged at 4°C. Serum was stored at −80°C for subsequent tests. Levels of total cholesterol (TC), high-density lipoprotein cholesterol (HDL-C), low-density lipoprotein cholesterol (LDL-C), triglycerides (TG), ALT, aspartate aminotransferase (AST), and gamma-glutamyl transpeptidase (GGT) were detected by standard enzymatic assays. White blood cells (WBC) and neutrophils were measured by automated hematology analyzer (SYSMEX XE-2100L, Japan). Fasting blood glucose (FBG) was measured using the glucose oxidase method, and fasting insulin (Fins) was measured using chemiluminescence. The formula for the homeostasis model assessment of insulin resistance (HOMA-IR) is as follows: HOMA-IR = FBG (mmol/L) × Fins (mU/L)/22.5.

### 2.3. Measurements of Serum PK2, IL-6, and TNF-*α*

Circulating PK2 concentrations were determined using a commercial ELISA kit according to the manufacturer's instructions (Catalog No. MBS2022546, MyBioSource, Inc., San Diego, USA). The detection range of the kit was 49.4–4000.0 pg/mL. The minimum detectable dose was 18.2 pg/mL, and the intra- and interassay coefficients of variation (CVs) were less than 10% and 12%, respectively. Serum IL-6 levels were assayed using commercial ELISA kits (Catalog No. CHE0009, Beijing 4A Biotech Co., Ltd, Beijing, China). The limit of detection was 2 pg/mL, and the intra- and interassay CVs were both less than 10%. Serum TNF-*α* levels were assayed using commercial ELISA kits (Catalog No. CHE0019, Beijing 4A Biotech Co., Ltd, Beijing, China). The limit of detection was 7 pg/mL, and the intra- and interassay CVs were both less than 10%.

### 2.4. Statistics

Statistical analyses were performed using the Statistical Package for Social Sciences (SPSS) version 21.0. Chi-square tests were used to analyze differences between categorical variables. Data distributions were determined using the Kolmogorov–Smirnov test. Continuous variables were presented as mean and standard deviation if they were normally distributed or as median with interquartile range if nonnormally distributed. Parametric tests (unpaired *t*-test) were used to assess differences between continuous variables if the distribution was normal, while nonparametric tests (Mann–Whitney *U* test) were used if the distribution was nonnormal. Spearman correlation analyses were performed to determine the associations between serum PK2 levels and other variables. Multiple linear stepwise regression analysis was performed to determine variables that were independently correlated with serum PK2 levels. The association of PK2 with obesity was examined by binary logistic regression analysis. Receiver operating characteristic (ROC) curves were used to analyze the predicting values of circulating PK2 for obesity. For all statistical tests, *P* *<* 0.05 were considered to indicate statistical significance.

## 3. Results

### 3.1. The Clinical and Laboratory Characteristics of All Subjects

There was no significant difference in the distribution of age and sex among the study subjects. The clinical and laboratory characteristics of the two groups are reported in Table 1. Compared with the control group, BMI, BMI z-score, Fins, FBG, HOMA-IR, TC, LDL-C, ALT, GGT, WBC, and neutrophil count were remarkably increased in the children with obesity. There was no significant intergroup difference in serum levels of TG, HDL-C, AST, IL-6, and TNF-*α* (Table 1). Furthermore, children with obesity with NAFLD had higher BMI, Fins, HOMA-IR, TG, ALT, AST, GGT, and TNF-*α* but lower HDL-C levels than children with obesity but without NAFLD (Table 2).

### 3.2. Comparison of Serum PK2 Levels between Different Groups

**Figure 1:**
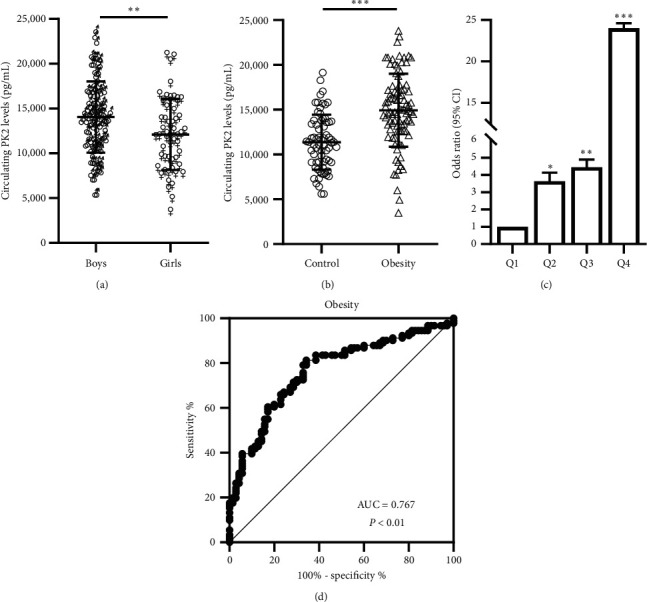
Circulating PK2 levels and ROC curve analysis in the study cohort. (a) Circulating PK2 levels in boys and girls (vs. boys:  ^*∗∗*^*P* < 0.01). (b) Circulating PK2 levels in children with obesity and normal-weight controls (vs. control:  ^*∗∗∗*^*P* < 0.001). (c) Prevalence of elevated obesity in different quartiles of PK2: quartile 1, <10654.9 pg/mL; quartile 2, 10654.9–13540.1 pg/mL; quartile 3, 13540.1–15857.4 pg/mL; quartile 4, >15857.4 pg/mL (vs. quartile 1:  ^*∗*^*P* < 0.05,  ^*∗∗*^*P* < 0.01,  ^*∗∗∗*^*P* < 0.001). (d) ROC curve analyses for the prediction of obesity according to the PK2 levels.

In the entire cohort, fasting serum PK2 levels were significantly higher in boys than girls (14054.4 ± 3975.9 vs. 12103.0 ± 3989.0 pg/mL, *P* *<* 0.01; Figure 1(a)). However, covariance analysis showed that children with obesity had elevated levels of fasting PK2 compared to the normal-weight controls (14937.2 ± 4073.6 vs. 11373.5 ± 3059.5 pg/mL, *P* *<* 0.001; Figure 1(b)) even after adjusting for sex. There was no significant difference in serum PK2 levels between children with obesity, with and without NAFLD.

### 3.3. Associations between Serum PK2 Levels and Metabolic Parameters

We performed Spearman correlation analysis to explore the relationship between serum PK2 concentrations and other parameters. The correlation analyses showed that serum PK2 levels were positively correlated with BMI, BMI z-score, Fins, FBG, HOMA-IR, TC, LDL-C, ALT, and GGT (Table 3). After controlling for sex, serum PK2 level was still positively associated with BMI, BMI z-score, Fins, FBG, HOMA-IR, TC, and LDL-C (Table 3). The multiple stepwise regression analysis showed that the main determinant of circulating PK2 was BMI. The beta-coefficient for BMI was 0.366 (Table 3).

To further investigate the relationship between PK2 and obesity, serum PK2 levels were divided into four quartiles according to its concentration in the entire cohort (quartile [Q] 1, <10654.9 pg/mL; Q2, 10654.9–13540.1 pg/mL; Q3, 13540.1–15857.4 pg/mL, and Q4, >15857.4 pg/mL). Then, binary logistic regression analysis was performed to calculate the odds ratio of obesity. As predicted, most individuals in the control group were in Q1 (57.1%), followed by 15.7% in Q2 and 21.4% in Q3; only a small proportion of subjects were in Q4 (5.7%). However, the percentage of children with obesity was highest in Q4 (39.5%), and the proportion in Q3 (27.5%) was also higher than in the Q1 (16.5%) and Q2 (16.5%) (Table 4). We found that the odds ratios for obesity were significantly elevated along with increasing PK2 quartiles (Figure 1(c)). For example, odds of having obesity in this group of children with the maximum level of PK2 (Q4) were 24 times as high as it was in the group of children with the minimum level of PK2 (Q1) (Table 4).

### 3.4. Receiver Operating Characteristic Analysis for Obesity

To further elucidate the relationship between PK2 and obesity, we performed ROC curve analysis of serum PK2 levels for predicting obesity. The area under ROC curves was 0.767 (*P* *<* 0.001) with a sensitivity of 81.3% and specificity of 65.7% for predicting obesity (Figure 1(d)). The best cut-off value for PK2 to predict obesity was 11930.0 pg/mL.

## 4. Discussion

In this study, we investigated the biochemical characteristics of fasting serum PK2 levels in normal-weight controls and children with obesity and analyzed the PK2 levels in children with obesity, with and without NAFLD. To our knowledge, this is the first study to investigate the circulating PK2 levels in children with obesity, with and without fatty liver disease.

In this research, compared with the normal-weight controls, children with obesity showed markedly increased serum PK2 levels. To date, two studies with adult patients have been performed to detect the level of PK2 in obesity-related metabolic diseases [25, 26]. Yong et al. showed that increased PK2 was independently associated with metabolic syndrome in a middle-aged and elderly Chinese population, and PK2 was positively correlated with FBG, blood pressure, and BMI [25]. However, Mortreux et al. showed that plasma PK2 was negatively correlated with BMI or energy intake in subjects with T2D [26]. The cause of the difference between the previous results and ours is unclear. These discrepancies could be attributed to (i) the different clinical characteristics of the different study cohorts, including age and distribution of sex, (ii) children with obesity having no other major complications such as vascular lesions and glucose metabolism disorders, and (iii) the difference in measurement, which may provide different results for PK2 levels. Szatkowski et al. demonstrated that PK2 levels were increased in the white adipose tissues of adult subjects with obesity, indicating PK2 may act as an adipose factor [23]. Nevertheless, we are yet uncertain regarding the reasons for the elevated PK2 expression and secretion in the present study. Besides, PK2 is controlled by a circadian clock [33]. When we asked the subjects or their legal guardians, none of them reported significant sleep and circadian rhythm abnormalities. Therefore, we speculate that high levels of PK2 in circulation, which are not mainly caused by impaired circadian rhythm, may reflect a compensatory response underlying metabolic stress. However, the direct role of PK2 in metabolic diseases still needs to be further investigated in future studies.

Our results found no statistical significance of circulating PK2 in children with obesity, with and without NAFLD. In the entire cohort, serum PK2 levels were positively correlated with BMI. In addition, BMI had significantly higher levels in children with obesity with NAFLD than in those without NAFLD. IL-6 and TNF-*α* were associated with a slightly higher grade of inflammation in children with obesity than the normal-weighted controls; however, this was not statistically significant. Previous studies showed that PK2 was related to inflammation [19, 20], whereas our results found that PK2 had no correlation with IL-6, TNF-*α*, and WBC and neutrophil count. It is likely that the increase of PK2 might only have been observed under acute inflammatory state, but this merits further investigation. We speculate that high levels of PK2 in patients with obesity may be correlated with BMI rather than the presence of NAFLD or inflammatory status. Nevertheless, these preliminary findings need confirmation in a larger sample size and further studies.

Human studies researching PK2 levels and insulin resistance are limited. In this study, circulating PK2 levels were positively correlated with HOMA-IR in the entire cohort. In patients with obesity, insulin signal transduction is impaired, leading to insulin resistance and increased HOMA-IR levels. PK2 can bind on the G protein-coupled receptor, namely, PKR1 [34]. We believe that PK2 increase might be a compensatory mechanism to elevate PK2/PKR1 signaling, which is known to suppress appetite, reduce adipocyte expansion, promote normal fat storage, and increase insulin sensitivity [35]. However, the association between PK2 and insulin resistance needs further research.

To further study the association between PK2 and obesity, we analyzed the prevalence of obesity in different quartiles of circulating PK2 levels. The results showed that the relative risks for obesity were markedly increased along with elevated PK2 levels. The children with the highest quartiles of PK2 concentrations had ∼24-fold higher risk of obesity than the children with the lowest quartile. Last, we analyzed the correlation of serum PK2 with obesity by means of the ROC curve. The data revealed that circulating PK2 might be a useful marker for the prediction of obesity.

Our study has some limitations. First, the current study is a cross-sectional study and does not demonstrate a causal relationship between increased circulating PK2 levels and the development of obesity. Second, our data could be influenced by some outliers owing to the relatively small sample size. Third, the individuals in this study were largely locally recruited, and the population mostly belongs to the Chongqing district and is of the Han ethnic group. Thus, future studies with larger sample sizes detecting PK2 in various conditions and populations will be needed.

## 5. Conclusion

In conclusion, our data provide clinical evidence that the elevation of serum PK2 may have a potential role in the pathogenesis of obesity, and it may be related to metabolic disorders and insulin resistance. In addition, the association between serum PK2 concentrations and BMI suggests that PK2 can be potentially used as a circulating biomarker for risk prediction of obesity. This is a pilot study, and a follow-up study is needed to investigate whether PK2 is a novel therapeutic target for obesity or obesity-related metabolic diseases, such as NAFLD and T2DM.

## Figures and Tables

**Table 1:** Comparison of clinical and laboratory parameters between control group and obese group.

Parameters	Control group (*n* = 70)	Obese group (*n* = 91)	*P* value
Gender (M/F)	46/24	60/31	0.977
Age (year)	9.01 ± 2.72	9.75 ± 2.50	0.079
BMI (kg/m^2^)	16.22 ± 1.83	27.13 ± 3.80	**<0.001**
BMI z-score	−0.15 ± 0.79	2.75 ± 1.08	**<0.001**
Fins (mU/L)	13.40 (7.55–18.13)	24.90 (13.40–35.90)	**<0.001**
FBG (mmol/L)	4.95 ± 0.32	5.28 ± 0.48	**<0.001**
HOMA-IR	2.79 (1.63–3.99)	6.01 (3.62–8.51)	**<0.001**
TG (mmol/L)	1.29 ± 0.48	1.37 ± 0.58	0.354
TC (mmol/L)	3.79 ± 0.56	4.10 ± 0.78	0.005
HDL-C (mmol/L)	1.29 ± 0.34	1.26 ± 0.27	0.568
LDL-C (mmol/L)	1.92 ± 0.51	2.52 ± 0.73	**<0.001**
ALT (U/L)	15.0 (12.0–17.3)	33.1 (21.3–55.1)	**<0.001**
AST (U/L)	29.0 (23.8–32.0)	26.0 (23.5–33.3)	0.723
GGT (U/L)	9.0 (7.0–11.0)	20.8 (15.4–32.0)	**<0.001**
Prokineticin 2 (pg/mL)	11373.5 ± 3059.5	14937.2 ± 4073.6	**<0.001**
IL-6 (pg/mL)	4.67 (3.30–6.61)	5.20 (1.71–8.10)	0.692
TNF-*α* (pg/mL)	6.67 (3.17–12.00)	9.35 (4.06–14.65)	0.072
WBC (×10^9^/L)	7.04 ± 1.43	7.82 ± 2.21	0.011
Neutrophils (×10^9^/L)	3.79 ± 0.91	4.42 ± 1.67	0.005

Data are shown as means ± SD or median (interquartile range). BMI, body mass index; Fins, fasting insulin; FBG, fasting blood glucose; HOMA-IR, the homeostasis model assessment of insulin resistance; TG, triglyceride; TC, total cholesterol; HDL-C, high-density lipoprotein cholesterol; LDL-C, low-density lipoprotein cholesterol; ALT, alanine aminotransferase; AST, aspartate aminotransferase; GGT, gamma-glutamyl transpeptidase; IL-6, interleukin-6; TNF-*α*, tumor necrosis factor-*α*; WBC, white blood cells.

**Table 2:** Comparison of clinical and laboratory parameters between obese group with or without NALFD.

Parameters	Without NAFLD group (*n* = 69)	With NAFLD group (*n* = 22)	*P* value
Gender (M/F)	43/26	17/5	0.197
Age (year)	9.52 ± 2.61	10.45 ± 2.02	0.128
BMI (kg/m^2^)	26.48 ± 3.65	29.18 ± 3.60	**0.003**
BMI z-score	2.71 ± 1.10	2.88 ± 1.05	0.536
Fins (mU/L)	23 (13.35–30.90)	34.40 (23.73–49.38)	**0.002**
FBG (mmol/L)	5.25 ± 0.44	5.35 ± 0.61	0.401
HOMA-IR	5.60 (3.08–7.25)	7.82 (5.96–12.04)	**0.001**
TG (mmol/L)	1.26 ± 0.55	1.60 ± 0.62	**0.016**
TC (mmol/L)	4.11 ± 0.74	4.02 ± 0.92	0.785
HDL-C (mmol/L)	1.30 ± 0.28	1.14 ± 0.21	**0.010**
LDL-C (mmol/L)	2.51 ± 0.72	2.46 ± 0.78	0.949
ALT (U/L)	27.6 (18.5–38.3)	60.9 (43.2–112.5)	**<0.001**
AST (U/L)	25.2 (23.0–31.0)	42.5 (25.7–61.4)	**<0.001**
GGT (U/L)	18 (14.9–24.5)	36.3 (30.5–70.1)	**<0.001**
Prokineticin 2 (pg/mL)	14885.4 ± 4145.8	15099.6 ± 3927.2	0.831
IL-6 (pg/mL)	5.20 (1.53–7.87)	5.49 (2.17–10.87)	0.974
TNF-*α* (pg/mL)	8.18 (3.47–12.29)	12.59 (7.88–17.15)	**0.030**
WBC (×10^9^/L)	7.75 ± 2.29	8.06 ± 2.00	0.563
Neutrophils (×10^9^/L)	4.41 ± 1.78	4.47 ± 1.31	0.890

Data are shown as means ± SD or median (interquartile range). BMI, body mass index; Fins, fasting insulin; FBG, fasting blood glucose; HOMA-IR, the homeostasis model assessment of insulin resistance; TG, triglyceride; TC, total cholesterol; HDL-C, high-density lipoprotein cholesterol; LDL-C, low-density lipoprotein cholesterol; ALT, alanine aminotransferase; AST, aspartate aminotransferase; GGT, gamma-glutamyl transpeptidase; IL-6, interleukin-6; TNF-*α*, tumor necrosis factor-*α*; WBC, white blood cells.

**Table 3:** Correlation analysis between prokineticin 2 and biochemical data in all participants.

Variables	PK2		PK2 (gender-adjusted)		Multivariate	
	*r*	*P*	*r*	*P*	*b*	*P*
Age	0.059	0.460	0.035	0.662	—	—
BMI	0.357	**<0.001**	0.357	**<0.001**	0.366	**<0.001**
BMI z-score	0.277	**0.001**	0.307	**<0.001**	—	—
Fins	0.389	**<0.001**	0.311	**<0.001**	—	—
FBG	0.253	**0.001**	0.214	**0.007**	—	—
HOMA-IR	0.382	**<0.001**	0.298	**<0.001**	—	—
TG	0.008	0.923	0.001	0.992	—	—
TC	0.171	**0.030**	0.165	**0.037**	—	—
HDL-C	0.039	0.620	0.009	0.915	—	—
LDL-C	0.233	**0.003**	0.209	**0.008**	—	—
ALT	0.349	**<0.001**	0.151	0.056	—	—
AST	0.020	0.804	0.069	0.383	—	—
GGT	0.401	**<0.001**	0.108	0.176	—	—
IL-6	−0.100	0.208	−0.040	0.613	—	—
TNF-*α*	−0.138	0.080	−0.137	0.084	—	—
WBC	0.090	0.258	0.115	0.148	—	—
Neutrophils	0.068	0.388	0.114	0.149	—	—

In the multiple linear stepwise regression analysis, the values included for analysis were BMI, Fins, FBG, LDL-C, ALT, GGT.

**Table 4:** Distribution of patients according to PK2 quartile.

PK2 quartile	Control group	Obese group	OR (95% CI)	*P* value
Q1	57.2%	16.5%	1	—
Q2	15.7%	16.5%	3.64 (1.37–9.67)	0.01
Q3	21.4%	27.5%	4.44 (1.86–10.64)	0.001
Q4	5.7%	39.5%	24.00 (7.29–78.99)	<0.001

Data are shown as percentages of the total number in each group according to PK2 quartile. OR denotes odds ratio of obese children compared with the group of children with the lowest ranges of concentration of PK2; the likelihood of obesity increases as the level of serum concentration of PK2 increases. PK2, prokineticin 2; Q, quartile; OR, odds ratio; CI, confidence ratio.

## Data Availability

The datasets generated during and/or analyzed during the current study are available from the corresponding author on reasonable request.

## Supplementary Materials

Supplementary Materials STORBE checklist for manuscript.
